# Quality management in the COVID-19 pandemic: nursing action plan

**DOI:** 10.1590/0034-7167-2022-0272

**Published:** 2023-02-03

**Authors:** Fabiana Aparecida Corrêa de Oliveira Braga, Sílvia Maria de Sá Basilio Lins, Bárbara Pompeu Christovam, Odilon Adolfo Branco de Souza

**Affiliations:** I Universidade Federal Fluminense. Niterói, Rio de Janeiro, Brazil

**Keywords:** Quality Management, Nursing, COVID-19, Pandemic, Tertiary Health Care., Gestión de la Calidad, Enfermería, COVID-19, Pandemia, Atención Terciaria de Salud., Gestão da Qualidade, Enfermagem, COVID-19, Pandemia, Atenção Terciária à, Saúde.

## Abstract

**Objectives:**

to describe the implementation of a nursing action plan to face the pandemic of COVID 19 in a University Hospital in the state of Rio de Janeiro**. Methods:** this is an experience report, in which two management tools were used: the Ishikawa Diagram to identify problems and the 5W2H spreadsheet to outline the actions according to the situations presented.

**Results:**

four categories were listed in two groups: 1- Actions of the Nurse Manager in the Organization of Logistics, Infrastructure and Care: materials and environment; and 2- Nursing Human Resources Management and Continuing Education: method and human resources.

**Final Considerations:**

the plan developed showed the role of nursing management, with the search for best care practices, development of protocols, carrying out multi-professional training and management of supplies.

## RESUMO

**Objetivos:**

descrever a implementação de um plano de ação da enfermagem para enfrentamento da pandemia de COVID-19 em um hospital universitário do estado do Rio de Janeiro.

**Métodos:**

trata se de um relato de experiência, no qual foram utilizadas duas ferramentas de gestão: o Diagrama de Ishikawa, para o levantamento dos problemas; e a planilha 5W2H, para traçar as ações de acordo com as situações apresentadas.

**Resultados:**

foram elencadas quatro categorias em dois grupos: 1) Atuação do enfermeiro gestor na organização de logística, infraestrutura e assistência: materiais e meio ambiente; e 2) Gerenciamento dos recursos humanos de enfermagem e educação permanente: método e recursos humanos.

**Considerações Finais:**

o plano elaborado evidenciou o protagonismo da gestão de enfermagem, com busca pelas melhores práticas assistenciais, elaboração de protocolos, realização de treinamentos multiprofissionais e gestão de insumos.

## RESUMEN

**Objetivos:**

describir la implementación de un plan de acción de enfermería para enfrentamiento de la pandemia de COVID-19 en un hospital universitario del estado de Rio de Janeiro.

**Métodos:**

tratase de un relato de experiencia, en lo cual fueron utilizadas dos herramientas de gestión: el Diagrama de Ishikawa, para el levantamiento de los problemas; y la hoja de cálculo 5W2H, para seguir las acciones de acuerdo con las situaciones presentadas.

**Resultados:**

fueron enumeradas cuatro categorías en dos grupos: 1) Actuación del enfermero gestor en la organización de logística, infraestructura y asistencia: materiales y medio ambiente; y 2) Gestión de los recursos humanos de enfermería y educación permanente: método y recursos humanos.

**Consideraciones Finales:**

el plan elaborado evidenció el protagonismo de la gestión de enfermería, con búsqueda por las mejores prácticas asistenciales, elaboración de protocolos, realización de entrenamientos multiprofesionales y gestión de insumos.

## INTRODUCTION

In 2020, modern nursing completed 200 years. Its protagonism was present not only in the celebrations but also in the actions to control and combat the disease caused by the novel SARS CoV 2 (Severe Acute Respiratory Syndrome Coronavirus 2), which puts public health worldwide at risk^(1)^. The global scenario is a fight and confrontation against the disease, which has spread and disorganized the health system and the economy, demanding immediate decisions and several hospital adaptations to contain its dissemination.

Therefore, strategies to assist the user in a timely and appropriate manner were imperative for hospital management. The updated epidemiological data was fundamental for the organization of the services, according to peaks of COVID-19 identified in the country and the region of the state of Rio de Janeiro (RJ). Such data subsidize the response to the challenges imposed on local management with prompt decisions to equip the institutions with supplies, equipment, trained human resources, and the creation of interventions and contingency plans in varied scenarios.

In this context, based on quality management, it is essential to maintain a high standard of care, in which everyone involved in health care is concerned with and closely linked to the constant improvement of the practices, in addition to focusing on the satisfaction of the service user. That standard of care is one of the defining concepts of quality management^(2)^.

In the epidemiological context of several cases of COVID-19 and with the administrative and structural difficulties that the hospital has experienced, to achieve the expected standard of quality care, it was necessary to employ techniques to define, measure, analyze and propose solutions to the problems that interfere with the performance of work processes - such techniques are called “quality tools^(2)^”.

There are many tools used in quality management. However, two are widely used for elaborating action plans: the Ishikawa Diagram and the 5W2H.

The Ishikawa Diagram, also known as fishbone, consists of a diagram whose purpose is to organize the reasoning in discussions of processes to achieve a priority problem. It can identify possible causes of a given situation, facilitating a more critical analysis, visualization, and interpretation^(2)^.

The 5W2H tool establishes simple and well-defined steps for a qualified and structured action plan, which maps out and standardizes processes through questions that guide action planning, such as: What will be executed? Where will it be carried out? When will it be carried out? Who will execute it? Why will the action be carried out? How will it be executed?^(2)^.

In this context, this experience report had as its object of study the development of an action plan with the application of managerial tools to provide the Nursing Division with subsidies for structuring organizational actions and work processes to confront the pandemic.

The present study indicates, as a knowledge gap, the management’s inability to solve the communication problem due to the impossibility of presential meetings since the pandemic took over the hospital’s daily routine without providing structured planning. An administrative vulnerability was identified; therefore, other studies are necessary to map the strategic-organizational actions that enabled behavioral and administrative changes resulting from the pandemic event.

## OBJECTIVES

To describe the implementation of an action plan to confront the COVID-19 pandemic in a university hospital in Rio de Janeiro.

## METHODS

The Ishikawa Diagram and the 5W2H were used to elaborate the action plan because those quality tools are among the five most used in Brazil and are part of the work context of nursing management professionals^(3)^.

The institutional flowchart of the investigated hospital comprises the Superintendence and, under it, the Health Care Management, Administrative Management, and Teaching and Research Management. Immediately underneath are the Nursing Division, Medical Division, and Diagnostic Support Division. Then, there are the nursing managers of five large areas: Surgical, Clinical, High Complexity, Maternal and Child Unit, and Emergency. Finally, below those managers, there are the assistance coordinators, responsible for each care unit.

The decree of the pandemic forced the hospital’s governance and its managers to establish a working group to elaborate a macro contingency plan. However, this experience report is limited to describing the actions of the Nursing Division and how it was structured to ensure that the decisions could reach the nurse coordinators responsible for providing care to the users.

Initially, weekly meetings were held between the head of the Nursing Division and the nursing managers. At these opportunities, they would discuss the nursing action plan for elaboration, adequacy, and alignment of actions. It had a bidirectional utility to support the decisions of the governance contingency plan, as well as to reach the nursing coordinators for knowledge and execution of the actions under their responsibility.

Between March 16 and May 31, 2020, six meetings were held with an average duration of four hours each. After discussing the processes and using the Ishikawa Diagram, they arrived at the priority problem established from two groups of correlated issues. Subsequently, they developed the nursing action plan using the 5W2H tool, with all actions debated and defined by consensus among the meetings’ participants.

Aiming to execute the proposed actions, these were disseminated among the hospital’s health teams, especially the care coordination, by promoting it during the day and night shifts through electronic communication channels.

## RESULTS

**Figure 1:**
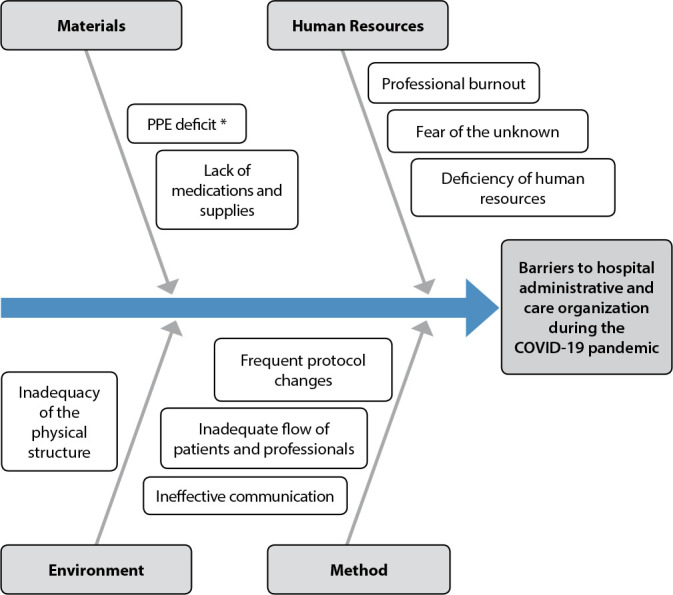
Problem raising using the Ishikawa Diagram, Niterói, Rio de Janeiro, Brazil, 2020

The priority problem raised in the meetings, originating from the application of the Ishikawa Diagram, was barriers for the administrative and care organization of the hospital during the COVID-19 pandemic. The first two categories discussed by the group were “Materials” and “Environment,” generating the “Group 1 - Actions of the nurse manager in the organization of logistics, infrastructure, and care”; the other two were “Methods” and “Human resources,” which generated the “Group 2 - Nursing human resources management and continuing education.” (Figure 1).

* *PPE - personal protection equipment.*

Figure 1 was divided into four categories already suggested by the tool: 1) Method - Insertion of problems related to the work method and current flows; 2) Materials - in this category, inclusion of problems related to medical-hospital equipment and supplies; 3) Environment - raising issues related to the physical structure and its inadequacies for the care of COVID-19 patients; and 4) Human Resources: inclusion of problems related to people, hospital professionals.

After surveying the problems listed in Figure 1, we used a new tool for resolution, the 5W2H, represented in Chart 1.

**Chart 1:** Action plan with the 5W2H tool, Niterói, Rio de Janeiro, Brazil, 2020

What	Why	Where	When	Who	How	How much
**ORGANIZATION OF LOGISTICS, INFRASTRUCTURE AND ASSISTANCE**						
Control of medical supplies and medications	Market shortage and inflated prices	In the whole hospital	During the first year of the pandemic, especially the first six months	Assistance Coordinators	Through evidence-based contingency plan of utilization	Unestimated Expense
Availability of a nurse to monitor the materials (standardization of PPE^*^)	To keep track of the quality and quantity required for material assistance	Warehouse and Purchasing Sector	In the acquisition of materials for assistance use	Nurse in partnership with the administrative sector	Through the assessment of the real needs for usage in the sectors and the product’s quality opinion	Unestimated Expense
Adequacy of the number of beds to the necessary distance and quantity of nursing professionals	Mandatory distance of 2 meters between beds	In all locations with inpatients	During the entire period	Assistance Coordinators	Reducing the number of beds	Unestimated Expense
**CONTINUING EDUCATION AND HUMAN RESOURCES**						
Organization with the University rectory of transportation for the hospital’s personnel and hotel accommodations	Allow transport of the health teams for care and accommodate the professionals	Several daily routes were established for the professionals’ attendance	During the lockdown period at some locations	University Drivers and Nursing Division	Routes established by necessity demands, due to transportation restriction	Unestimated Expense
Request for emergency professionals to attend COVID-19 to the competent body	Adequate the COVID dimensioning, established by COFEN† and compensate for the exit of vulnerable professionals	In all sectors intended for COVID-19 service	During the pandemic	Nursing Division and Hospital Superintendent	Reports demonstrating the need for personnel	Unestimated Expense
Provide effective communication between the nursing teams	Spreading the knowledge of the elaborated processes and the changes in the assistance protocols	Inside the hospital	After each decision making with changes in routine and care processes	Team managers and nurse coordinators	Through registrations in order books and sectoral occurrences, electronic mail, and social networks	Unestimated Expense
Organization of incoming and outgoing flows of professionals and patients	Avoid cross flow between suspected and contaminated patients, with professionals and other patients	In the COVID-19 service sectors and outpatient areas	During the pandemic	Risk Management Team, HICC‡ and Nursing Division	Signs on the floor with green and red stripes and seats inside the hospital	Unestimated Expense

* PPE - personal protection equipment;

† COFEN - Federal Nursing Council;

‡ HICC - Hospital Infection Control Committee.

Chart 1 represents an action strategy to organize which activities will be developed, why they are listed, who will execute them, when they will be executed, how they will be executed, and what the cost of the action will be. In this way, it is possible to control what has been accomplished, which professional would do it, and how those actions are progressing within the planning.

## DISCUSSION

The hospital’s action plan was based on global and local scientific evidence, mainly on the guidelines of the Ministry of Health, such as the National Contingency Plan for Human Novel Coronavirus Infection (COVID-19), from February 2020^(4)^. Thus, we compiled into groups the actions developed to confront the pandemic.

In Group 1 (Performance of the nurse manager in the organization of logistics, infrastructure, and care), specifically in the category “Material,” in the Ishikawa diagram, there was a deficit of PPE and a lack of medications and supplies. The Ministry of Health established as a control measure the guarantee of supplies for diagnosis and treatment of human infection by the novel coronavirus and other respiratory viruses for the laboratory and healthcare network; however, the supplies had increased in value and became scarce in 2020^(2,4)^. The management monitored the quantity utilized in the sectors and evaluation of usage peaks for 24 hours, with the nurse supervisor participating in the inspection and orientation for that usage.

Technical Note 4/2020 recommends the extended use of PPE^(5)^. Technical Note 2/2020, on the other hand, suggests that the pharmaceutical, health products, cosmetics, and sanitizing industries make rational use of disposable respirators (N95 masks) and, whenever possible, donate the surplus units to health services^(6)^. That has enabled the management to avoid shortages by reorganizing the purchase and utilization of those supplies.

The increase in drug consumption raised monthly expenses, emphasizing the drugs that most impacted the Hospital Drug Price Index (IPM-H) during the pandemic: norepinephrine; fentanyl; propofol; midazolam; omeprazole; and pantoprazole. Among the main reasons for the rise in this index is the devaluation and/or exchange rate variation, which drastically affects the price of medications, especially those imported^(2)^. Another significant and widely used supply, which also suffered a shortage, was the N95 mask. There was an exchange of items or loans and contingency in elective treatments to solve that problem.

Organizing the distribution and quality evaluation of the products was urgent at that moment, and it was necessary to assign nurses to attend to the standardization and distribution of the materials. To that end, were considered the guidelines for COVID-19 diagnosis and treatment and the sectorial demands. That way, it was created a list of the professionals who would use the N95 masks, the surgical masks, and the aprons. The disposable supplies (surgical masks, aprons) were distributed daily, considering the sector’s turnover for dispensing. The standardization and storeroom nurse was responsible for evaluating the quality of the delivered materials and monitoring, with the storekeeper, the demand for each service.

Still, in Group 1, the category “Environment,” in the Ishikawa Diagram, identifies the inadequacy of the physical structure caused by the emergence of the pandemic. With the event, it was necessary to adapt the hospital units to the implications of COVID-19 due to the need for isolation of some areas (cohorts) and the growing demand for hospital beds.

Brazil has 270,880 available beds (clinical and surgical) and 34,464 Adult Intensive Care Unit (ICU) beds, 66% and 48% available for the Unified Health System (SUS), respectively. The high number of small hospitals, 5,345 (66%), draws attention, of which 70% have up to 29 beds. Only 10% of the hospital establishments are large-sized (above 150 beds). Although fewer in number, those hospitals concentrate 42% of the beds, followed by the medium-sized ones (51 to 150 beds), with 35%. The occupation rate of available beds in the SUS is relatively low for small-sized hospitals, 24% (up to 29 beds) and 32% (between 30 and 50 beds), compared to 75% in large-sized hospitals. There is a more significant SUS depletion for ICU beds, especially in large hospitals, with an average occupancy rate of 60% (medium size) and 77% (large size)^(7)^. The actions listed for this problem are described below.

Among the measures adopted were the adaptation of the number of beds, cohorts in some sectors, expansion of the number of intensive care beds, and division of spaces into “COVID” and “non-COVID” areas with possible adaptation according to the peaks of the disease. Also, some infirmaries had to be closed to meet the distancing of beds. In the emergency service, it was created an exclusive area to attend to the patients with flu symptoms, a space for RT-PCR (Reverse Transcription - Polymerase Chain Reaction) collection, and a COVID-19 Critical Care Unit^(8)^.

In the presented scenario, it is urgent to deal with the issues addressed in Group 2 (Management of human resources for nursing and continuing education), in the category “Human Resources,” in the Ishikawa Diagram: fear of the unknown, professional burnout, human resources deficiency. In the context of the pandemic, the management of people assumes expressive importance given the work overload, occupational risks, and possibility of illness, in addition to the conditions mentioned above of insufficient and/or limited human resources.

It was organized, with the university rectory, the availability of transportation for the hospital’s employees to confront this reality. Given the lockdown of several municipalities in the state of Rio de Janeiro and the difficulty of access to work, the Nursing Division developed routes in partnership with the transportation teams to allow the transportation of employees and enable the continuity of care^(9)^. In addition, employees who did not feel safe returning home could stay in a university-sponsored hotel.

Insufficient human resources of various categories, especially the nursing team, often hindered the service or even prevented its continuity. With that, it was necessary to elaborate reports to demonstrate the reduction in the number of nursing professionals due to the absence of vulnerability and illness, either from family members or the professionals themselves.

The competent authority in Brasilia responded to the emergency by hiring approximately 200 nursing professionals to help the hospital during the pandemic. After hiring the personnel, the nursing management had to plan the balance of shifts through the reallocation of days and sectors and equalize the distribution between old and new professionals to update the routines and norms of the services.

Regarding the “Method” category in the Ishikawa Diagram, as frequent conduct changes, patient flow, and inadequate professionals, the Ministry of Health, on February 3, 2020, declared a public emergency through Ordinance No. 188. The institution recommended that state and municipal health departments and public and private services develop contingency plans with response measures proportional and specific to the current risks to reduce the chances of ongoing transmission and the emergence of severe cases with subsequent deaths^(10)^.

Frequent changes of conduct emerged, such as better care practices, given the variations presented by the virus and health professionals’ learning to manage and deal with the new reality. Thus, care protocols and standard operating procedures were elaborated to unify and disseminate technical and safe conduct to the user and the hospital’s professionals. In an effort to accomplish this dissemination process, it was used technology allied to education, based on the creation of educational videos and simulations in the work environment by the permanent education and patient safety nurses. In addition, the protocols, and contingency plans on how, who, and where to execute care and administrative procedures were published in electronic media and social networks.

With the need to create flows, nursing management, in partnership with hospital governance, risk management unit, and the Hospital Infection Control Committee (HICC), established flows to avoid cross-contamination, with the development of ways to disseminate to all nursing teams through e-mails and messages via social network. That’s because the care with the flow of patients suspected or confirmed with respiratory diseases and the rational use of personal protective equipment are essential points in the chain of disease dissemination, inside and outside the health services^(8)^.

The hospital access was limited to two entrances to establish the flow of people inside the institution: one for users at the main door and another for employees at the side door. Nursing professionals were strategically positioned at those entrances to provide guidance on flu symptoms, mask supply, hand hygiene with alcohol gel, and direct flows in green areas (free passage areas) and red areas (restricted areas for patients suspected or contaminated with COVID-19.

### Study limitations

The current study presents, as a limitation, the absence of a structured methodological framework, since it is an experience report. If there had been enough time, an action plan based on the referential of Implementation Science would ensure greater consistency to the content presented; however, the pandemic invaded the hospital reality without allowing any previous planning.

### Contributions to the field of Nursing

This study’s contribution is to guide the decision-making actions of the nursing team through available and known efficient management tools, enabling an adequate and specialized service for the treatment of patients suspected or confirmed with COVID-19.

## FINAL CONSIDERATIONS

A significant role of the nursing management teams in this pandemic context is operating in developing protocols, standard operating procedures, daily training with the teams, and searching for knowledge and evidence for better assistance to users. The pandemic demanded an enormous effort from hospital and nursing service managers. It challenged them to act and think of service flows promptly to apply scientific principles involving mobilization of professional, administrative, social, and emotional competence, aiming at the fast and efficient reception of this new demand for care.

The current scenario, resulting from COVID-19, imposed structural and process revisions, protocols, and routes in the inpatient units. It made the nurse manager, along with the teams (created in the nursing division’s action plan), understand the weaknesses of the nursing services and then review, build, and rebuild guidelines for an effective, identifiable, and applicable work process.

Therefore, it is evident the importance of continuing education in services and the description of the stages of training and development of protocols, aiming at the team’s safe work and the continuous learning of nursing teams, transforming knowledge, developing scientific research, and elaborating new knowledge. There is still much to learn from the changes imposed by the pandemic context. However, it is necessary to believe that, in the face of an adverse scenario, the nursing profession emerges as a primary anchor of the health service, bringing reflection about the importance of their role in society.
